# Association Between Contrast Sensitivity and Ganglion Cell–Inner Plexiform Layer Thickness After Resolution of Macular Edema Due to Branch Retinal Vein Occlusion

**DOI:** 10.3390/jcm14051507

**Published:** 2025-02-24

**Authors:** Tomoya Murakami, Fumiki Okamoto, Takeshi Matsueda, Yoshimi Sugiura, Shohei Morikawa, Yoshifumi Okamoto, Takahiro Hiraoka, Tetsuro Oshika

**Affiliations:** 1 Department of Ophthalmology, Faculty of Medicine, University of Tsukuba, Tsukuba 305-8577, Japan; edatake909@gmail.com (T.M.); yoshimis@md.tsukuba.ac.jp (Y.S.); shomorikawa@md.tsukuba.ac.jp (S.M.); y-okamoto@md.tsukuba.ac.jp (Y.O.); thiraoka@md.tsukuba.ac.jp (T.H.); oshika@eye.ac (T.O.); 2 Department of Ophthalmology, Graduate School of Medicine, Nippon Medical School, Tokyo 113-8603, Japan; f-okamoto@nms.ac.jp; 3 Department of Ophthalmology, Mito Kyodo General Hospital, Mito 310-0015, Japan

**Keywords:** branch retinal vein occlusion, contrast sensitivity, ganglion cell, optical coherence tomography

## Abstract

**Background/Objectives:** We sought to assess the relationship between contrast sensitivity (CS) and optical coherence tomography (OCT) findings, including ganglion cell–inner plexiform layer (GCIPL) thickness, in eyes with cystoid macular edema, secondary to branch retinal vein occlusion (BRVO-CME), treated with intravitreal ranibizumab (IVR). **Methods**: This prospective study included 44 patients with BRVO-CME who underwent treatment with IVR (three monthly injections and pro re nata) and were followed up for 12 months. We collected data on CS, best-corrected visual acuity (BCVA), and OCT findings (ellipsoid zone [EZ] and external limiting membrane status [ELM], central foveal thickness [CFT], and average GCIPL thickness) at the time of the final visit when macular edema was resolved. Multiple regression analysis was used to evaluate the relationship between visual functions and OCT findings, age, and lens status. **Results**: Multiple regression analysis revealed that lower GCIPL thickness was significantly associated with worse CS (β = 0.008; 95% CI, 0.002–0.014; *p* = 0.011), whereas this was not the case with BCVA. Lower CFT and mild cataracts were also associated with worse CS (CFT: β = 0.003; 95% CI, 0.001–0.004; *p* = 0.001; mild cataract: β = −0.182; 95% CI, −0.286–−0.078; *p* = 0.001) and worse BCVA (CFT: β = −0.002; 95% CI, −0.003–−0.001; *p* < 0.001; mild cataract: β = 0.079; 95% CI, 0.008–0.150; *p* = 0.029). **Conclusions**: GCIPL thickness may serve as a valuable biomarker for CS in eyes with BRVO-CME following IVR treatment.

## 1. Introduction

Branch retinal vein occlusion (BRVO) ranks as the second most prevalent disorder affecting the retinal blood vessels [1], with macular edema being the leading cause of vision impairment linked to BRVO [2]. The introduction of anti-vascular endothelial growth factor (VEGF) therapy has led to better visual outcomes [3,4]. However, some patients complain of unsatisfactory visual function, such as blurred vision, even after the complete resolution of macular edema.

Contrast sensitivity (CS) is a critical visual function and has been shown to be a significant predictor of visual performance in daily life [5]. Visual acuity (VA) testing evaluates the capability to distinguish between high-contrast letters of different sizes, whereas contrast sensitivity (CS) testing measures the capacity to detect details using letters at various contrast levels. In our daily lives, we encounter a variety of objects and scenes at different contrast levels. Therefore, contrast sensitivity assessments are more closely aligned with functional vision, applicable to everyday activities, than with VA tests.

Previous studies have evaluated CS in patients with retinal vein occlusion (RVO) following anti-VEGF treatment [6,7,8,9,10,11,12]. Silverman et al. reported that the CS of eyes treated for BRVO with anti-VEGF therapy was worse than that in age-matched controls [6]. Previously, we reported that contrast sensitivity (CS), rather than visual acuity (VA), was closely linked to vision-related quality of life (VR-QOL) in patients with central retinal vein occlusion (CRVO) who underwent anti-VEGF treatment [13]. Some investigators assessed the relationship between CS and VR-QOL in patients with retinal disorders other than RVO, and they reported that CS, rather than VA, was associated with VR-QOL in patients after retinal detachment surgery [14], and that changes in CS before and after treatment were significantly correlated with changes in VR-QOL in patients with PDR and DME [15]. Based on the results of previous studies, CS is considered an important visual function in patients with some vitreoretinal diseases. No study has reported on the relationship between CS and VR-QOL in patients with BRVO; however, CS might be one of the most important visual functions in patients with BRVO.

Optical coherence tomography (OCT) is a non-invasive imaging method that delivers high-resolution, cross-sectional views of the retina, choroid, and optic nerve with near-histological detail. By allowing clinicians to observe and measure various retinal layers in detail, OCT has become an essential tool, not only for diagnosing and managing eye diseases, but also for detecting systemic conditions that impact the retinal microstructure [16,17]. Its ability to accurately quantify retinal layer thickness and identify subtle structural alterations has transformed our understanding of retinal diseases and improved the evaluation of therapeutic outcomes. In cases of RVO, OCT instrumental in evaluating structural changes in the retinal layers. Alshareef et al. investigated changes in retinal ganglion cells in eyes with BRVO using OCT [18]. They reported a significant reduction in macular ganglion cell–inner plexiform layer (GCIPL) thickness in eyes with BRVO, suggesting that retinal ischemia and cotton wool spots may be responsible for GCIPL thinning. While GCIPL thinning in eyes with CRVO is associated with reduced contrast sensitivity after macular edema resolution [15], in eyes with BRVO, the relationship between GCIPL thickness and contrast sensitivity is not well known.

Therefore, in this study, we aimed to determine the relationship between various OCT findings, including GCIPL thickness and CS, in eyes with BRVO-CME after anti-VEGF therapy.

## 2. Materials and Methods

This was a multicenter, open-label, single-arm, prospective study. The Institutional Review Board of the Tsukuba University Hospital and Mito Kyodo General Hospital approved this study (number: H27-238; approval date: 26 February 2016). This study adhered to the tenets of the Declaration of Helsinki. We enrolled patients with BRVO-CME who were referred to the Tsukuba University Hospital or Mito Kyodo General Hospital between March 2016 and December 2018. The exclusion criteria were ophthalmic disorders in the affected or fellow eye, except for mild refractive errors and cataracts; severe diabetes; severe hypertension; cerebrovascular diseases; and ischemic heart diseases. Patients who had received anti-VEGF intravitreal injections, intravitreal injections, or subtenon triamcinolone acetonide (STTA) injections, intraocular surgery within the past 90 days, or retinal laser photocoagulation within the past 30 days were also excluded.

### 2.1. Assessments

We collected the data of CS and BCVA and analyzed the retinal structure using SD-OCT at baseline and at the time of the final visit upon the resolution of macular edema. The BCVA was assessed utilizing the Landolt chart, and the measurements were transformed into logarithms of the minimum angle of resolution (logMAR) values for analytical purposes.

CS was evaluated using the CSV-1000E chart (Vector Vision, Greenville, OH, USA), along with the optimal spectacle correction. This chart utilizes a fluorescent light source, set to a luminance of 85 cd/m^2^. It measures CS across four spatial frequencies (3, 6, 12, and 18 cycles per degree), offering eight contrast levels for each. The contrast level at which the last correct answer was given was documented as the contrast threshold, expressed in logarithmic terms. Furthermore, the area under the log contrast sensitivity function (AULCSF) was derived using the data obtained from the CSV-1000E chart.

We performed macular imaging using spectral domain optical coherence tomography (Cirrus high-definition OCT; Carl Zeiss AG, Jena, Germany), which included macular cube scans and five-line raster cross scans. Scans with a signal strength exceeding 6/10 were considered suitable. From the OCT images, we collected data on various OCT findings, including the status of the EZ and ELM, central foveal thickness (CFT), and the average thickness of the GCIPL. EZ and ELM statuses were categorized as intact or disrupted. The evaluation of EZ and ELM was conducted within a 1 mm area centered on the fovea in both vertical and horizontal OCT sections. The CFT at the foveal center was manually measured using Cirrus analysis software. The average GCIPL thickness was calculated using the macular cube image. Cases with segmentation errors, detected during ganglion cell analysis, were excluded. Two evaluators (TM and YS), who were blinded to patients’ clinical data, assessed all OCT findings. Discrepancies were resolved through consultation with a third blinded author (FO) until a consensus was reached.

In addition, we collected patient data including age and lens status, categorized as a clear lens, a mild cataract, or an intraocular lens (IOL).

### 2.2. Treatment

Patients received initial treatment consisting of three intravitreal injections of ranibizumab (IVR; 0.5 mg, Lucentis; Novartis Pharma K.K., Tokyo, Japan), administered monthly. Further IVR injections were given based on the following criteria for additional treatment: (1) CFT greater than 300 μm; (2) new or ongoing cystoid changes in the retina; (3) subretinal detachment; or (4) subretinal hemorrhage.

### 2.3. Analysis

We evaluated the changes in visual function (AULCSF and BCVA) and CFT with the Wilcoxon signed-rank test. To examine the association with visual function, we applied the unpaired *t*-test or Kruskal–Wallis test for categorical variables, and the Spearman rank correlation for the relationship of visual function with continuous variables. Multivariate analysis with stepwise regression was used to assess the relationship between visual functions and various variables (OCT findings, lens status, patients’ age). All analyses were performed with SPSS V.28, and a *p*-value of less than 0.05 was considered significant for all analyses.

## 3. Results

**Figure 1:**
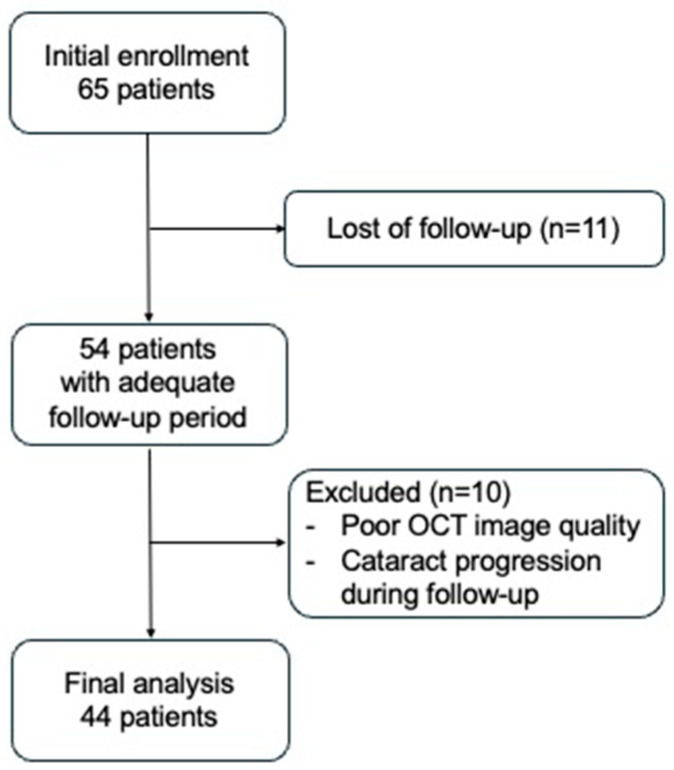
Flowchart of participant inclusion.

Overall, 44 eyes of 44 patients with BRVO were enrolled in this study (Figure 1). Three eyes were previously treated for BRVO (with laser treatment for 3 eyes, and STTA for 1 eye). None of the laser-treated eyes had received treatment within the measurement area of GCIPL, and so they were included in the analysis. In 27 eyes, macular edema was mostly resolved 12 months after treatment. In 12 eyes, macular edema was mostly resolved 11 months after treatment. In 5 eyes, macular edema was most resolved 7 to 10 months following treatment. No adverse events, such as cataract development, were observed after IVR. Table 1 shows baseline clinical characteristics, visual functions, and CFT at baseline and at time the macular edema was resolved.

Table 2 lists the relationship between AULCSF and OCT findings, age, and lens status at time the macular edema was resolved. The bivariable analysis revealed that the lens status, GCIPL thickness, CFT, and age were significantly associated with AULCSF (*p* = 0.010, *p* < 0.001, *p* < 0.001, *p* = 0.012, respectively). Multivariable analysis revealed that mild cataract and lower GCIPL thickness and CFT were significantly associated with worse AULCSF (*p* = 0.001, *p* = 0.011, *p* = 0.001, respectively).

Table 3 lists the relationship between BCVA and OCT findings, age, and lens status at the time the macular edema was resolved. The bivariable analysis revealed that the EZ status, GCIPL thickness, and CFT were significantly associated with BCVA (*p* = 0.014, *p* = 0.004, *p* < 0.001, respectively). The multivariable analysis revealed that mild cataracts and lower CFT were significantly associated with worse BCVA (*p* = 0.029, *p* < 0.001, respectively).

## 4. Discussion

In this study, multiple regression analysis revealed that the GCIPL thickness, CFT, and lens status were significantly associated with CS, and CFT and lens status were associated with BCVA in eyes with BRVO-CME following anti-VEGF therapy. We previously investigated the relationship between OCT findings and CS in patients with CRVO-CME following anti-VEGF therapy. We reported that GCIPL thickness was significantly associated with CS and BCVA in eyes with CRVO-CME at macular edema resolution [13], which is consistent with the result of this study. Several studies have examined the relationship between inner retinal structure and CS in RVO. Nixon et al. investigated the relationship between CS and ganglion cell volume in 25 eyes with RVO (11 CRVO and 14 BRVO) following anti-VEGF therapy [7] and reported that there was a weak correlation between these parameters. In contrast, our study demonstrated a moderate correlation between CS and GCIPL thickness (r = 0.500). The difference in correlation strength may be attributed to several methodological factors, including our larger sample size (44 eyes) and the specific exclusion of cases with segmentation errors in ganglion cell analysis. Wang et al. investigated the relationship between DRIL and CS in 58 eyes with RVO (29 eyes with BRVO, 29 eyes with CRVO), and they reported that DRIL was significantly associated with CS [9]. DRIL is thought to represent the disruption of the visual pathway through the dysfunction of bipolar, amacrine, and retinal ganglion cells [9]. Based on the results of this study and previous studies, we believed that retinal ganglion cell’s damage might have an important role in reducing CS in eyes with BRVO. Since visual acuity alone may not fully capture the extent of visual dysfunction in patients treated with anti-VEGF for BRVO, and as CS provides additional insights into visual performance, GCIPL thickness can serve as a valuable biomarker for assessing visual outcomes in these patients.

Several researchers investigated relationships between CS and GCIPL eyes with ocular diseases other than RVO, and they reported that CS was associated with GCIPL thickness in eyes with glaucoma [19] and multiple sclerosis [20,21]. Shamsi et al. studied the retinal layers associated with contrast sensitivity in 225 subjects, including individuals with either glaucoma, age-related macular degeneration, or normal vision, using deep learning [22]. They reported the ganglion cell and inner plexiform layers were the most critical layer linked to the CS, whereas the photoreceptor layer was most critical retinal layer linked to the visual acuity. Based on these previous reports, it can be inferred that healthy ganglion cells play a significant role in maintaining good CS.

Alshareef et al. reported that the GCIPL of eyes with BRVO was thinner than the normal control, and they asserted that ischemia caused by vein occlusion may lead to focal axonal transection and cause the descending ganglion cell’s degeneration and atrophy [18]. The ganglion cell’s degeneration and atrophy, caused by ischemia, might have an important role in poor visual function, such as CS reduction, in eyes with BRVO after the resolution of macular edema.

The multivariable analysis revealed that decreased CFT was associated with worse CS and BCVA results. It was reported that the decreased CFT was associated with worse BCVA [13,23,24] and CS [13] in eyes after the resolution of macular edema secondary to RVO. Based on the results of previous reports and this study, we believe that the damage or loss of photoreceptors at fovea have an important role in reduced CS and BCVA in eyes with RVO after the resolution of macular edema.

In this study, eyes with mild cataracts had worse VA and CS than those with clear lenses. It was reported that cataracts can reduce both BCVA and CS [25]. Wang et al. investigated factors associated with CS and BCVA in eyes with RVO, and they reported that both DRIL and lens status were significantly associated with CS and BCVA [9], which aligns with our study’s results. Lens status can be considered an important factors affecting CS and BCVA in eyes with BRVO after the resolution of macular edema.

The limitations of this study include its relatively smaller sample size and the lack of assessment of the degree of ischemia. It was reported that inner retinal ischemia contributes to vision loss in eyes BRVO [26]. Lee et al. investigated the relationship between GCIPL thickness and macular microcirculation using OCT and OCT–angiography in patients with type 2 diabetes [27] and found that GCIPL thickness was positively associated with vessel density. Based on these previous studies, inner retinal ischemia associated with retinal vascular disorders appears to result in damage to inner retinal neurons, particularly retinal ganglion cells, and we speculated that the inner retinal ischemia may contribute to GCIPL thinning, which consequently results in reduced CS in eyes with BRVO after the resolution of macular edema. If ganglion cell death due to ischemia is indeed the cause of CS reduction in BRVO, the neuroprotective agents currently being developed for retinal artery occlusion treatment might prevent CS deterioration in cases where severe ischemia is predicted based on initial fluorescein angiography findings and the presence of cotton wool spots [28]. From this perspective, further investigation of the relationship between ischemia, GCIPL thickness, and CS would be clinically significant. Further research with larger sample sizes to assess the degree of ischemia, using fluorescein angiography or OCT–angiography, is warranted.

## 5. Conclusions

Lower GCIPL thickness and CFT were associated with worse CS, and lower CFT was associated with worse BCVA in eyes with BRVO at the time of the resolution of macular edema. We considered that inner retina damage contributes to reduced CS in eyes with BRVO. In terms of CS, the GCIPL thickness may be an important biomarker in eyes with BRVO-CME.

## Figures and Tables

**Table 1:** Baseline clinical characteristics, visual functions, and CFT at baseline and at time the macular edema was resolved.

	Baseline	At the Time the ME Was Resolved	*p*
Age, years	66.4 ± 10.9		
Sex (male/female)	20/24		
Lens status (clear lens/mild cataract/IOL)	11/28/5		
AULCSF	0.75 ± 0.22	1.13 ± 0.22	<0.001 *
BCVA (logMAR)	0.39 ± 0.29	0.03 ± 0.13	<0.001 *
CFT (µm)	518 ± 210	190 ± 36	<0.001 *

CFT—central foveal thickness; ME—macular edema; IOL—intraocular lens; AULCSF—the area under the log contrast sensitivity function; BCVA—best-corrected visual acuity. * significant difference found between the parameters (Wilcoxon signed-rank test, * *p* < 0.001).

**Table 2:** The relationship between AULCSF and OCT findings, age, and lens status at the time the macular edema was resolved.

Figure		Bivariable Analysis		Multivariable Analysis		
	Number	Mean ± SD	*p*	β	95% Confidence Interval (Lower, Upper)	*p*
ELM disruption (+)	8	1.08 ± 0.18	0.514			
(−)	36	1.14 ± 0.23				
EZ disruption (+)	20	1.09 ± 0.20	0.098			
(−)	24	1.17 ± 0.23				
Clear lens	11	1.28 ± 0.16	0.010 *	Reference	Reference	
Mild cataract	28	1.06 ± 0.23		−0.182	−0.286, −0.078	0.001 ^‡‡^
IOL	5	1.09 ± 0.05				
		r	*p* value			
GCIPL thickness		0.500	<0.001 ^††^	0.008	0.002, 0.014	0.011 ^‡^
CFT		0.479	<0.001 ^††^	0.003	0.001, 0.004	0.001 ^‡‡^
Age		−0.346	0.021 ^†^			

AULCSF—the area under the log contrast sensitivity function; SD—standard deviation; ELM—external limiting membrane; EZ—ellipsoid zone; IOL—intraocular lens; GCIPL—ganglion cell–inner plexiform layer; CFT—central foveal thickness. * significant correlations found between the parameters (Kruskal–Wallis test: * *p* < 0.05). ^†^ significant correlations found between the parameters (Spearman rank correlation coefficient: ^†^ *p* < 0.05; ^††^ *p* < 0.001). ^‡^ significant correlations found between the parameters (multiple regression analysis: ^‡^ *p* < 0.05; ^‡‡^ *p* < 0.005).

**Table 3:** The relationship between BCVA and OCT findings, age, and lens status at the time the macular edema was resolved.

		Bivariable Analysis		Multivariable Analysis		
	Number	Mean ± SD	*p*	β	95% Confidence Interval (Lower, Upper)	*p*
ELM disruption (+)	8	0.10 ± 0.13	0.093			
(−)	36	0.01 ± 0.13				
EZ disruption (+)	20	0.08 ± 0.11	0.014 *			
(−)	24	−0.02 ± 0.14				
Clear lens	11	−0.01 ± 0.13	0.243	Reference	Reference	
Mild cataract	28	0.05 ± 0.14		0.079	0.008, 0.150	0.029 ^‡^
IOL	5	−0.03 ± 0.11				
		r	*p*			
GCIPL thickness		−0.427	0.004 ^†^			
CFT		−0.483	<0.001 ^††^	−0.002	−0.003, −0.001	<0.001 ^‡‡^
Age		0.214	0.162			

BCVA—best-corrected visual acuity; SD—standard deviation; ELM—external limiting membrane; EZ—ellipsoid zone; IOL—intraocular lens; GCIPL—ganglion cell–inner plexiform layer; CFT—central foveal thickness. * significant correlations found between the parameters (unpaired *t*-test: * *p* < 0.05). ^†^ significant correlations found between the parameters (Spearman rank correlation coefficient: ^†^ *p* < 0.005; ^††^ *p* < 0.001). ^‡^ significant correlations found between the parameters (multiple regression analysis: ^‡^ *p* < 0.05; ^‡‡^ *p* < 0.001).

## Data Availability

Study data are available from the corresponding author on reasonable request.
